# Prevalence and comorbidities of self-reported physician-diagnosed obesity among adults attending primary healthcare centers in Riyadh, Saudi Arabia: a cross-sectional survey

**DOI:** 10.3389/fpubh.2026.1755138

**Published:** 2026-03-20

**Authors:** Emad Aljohani, Mamdouh M. Shubair, Faris Fatani, Tahani Mubarak Alotaibi, Aljohrah Ibrahim Aldubikhi, Awad Alshahrani, Badr F. Al-Khateeb, Abdullah A. Albarrak, Nadia Mohamad Asiri, Ashraf El-Metwally

**Affiliations:** 1 Department of Surgery, College of Medicine, Prince Sattam Bin Abdulaziz University, Al Kharj, Saudi Arabia; 2 School of Health Sciences, University of Northern British Columbia (UNBC), Prince George, BC, Canada; 3 Riyadh Second Health Cluster, Riyadh, Saudi Arabia; 4 Ministry of Health, Deputy of Public Health, General Directorate of Health Programs and Chronic Conditions, Riyadh, Saudi Arabia; 5 College of Health Sciences, Saudi Electronic University, Riyadh, Saudi Arabia; 6 Ministry of the National Guard-Health Affairs, Riyadh, Saudi Arabia; 7 King Abdullah International Medical Research Center, Riyadh, Saudi Arabia; 8 College of Medicine, King Saud bin Abdulaziz University for Health Sciences, Riyadh, Saudi Arabia; 9 Department of Surgery, College of Medicine, Majmaah University, Al-Majmaah, Saudi Arabia; 10 Department of Health Education, Al Kharj Armed Forces Hospitals, Al Kharj, Saudi Arabia; 11 College of Public Health and Health Informatics, King Saud Bin Abdulaziz University for Health Sciences, Riyadh, Saudi Arabia

**Keywords:** comorbidities, obesity, predictors, prevalence, Saudi Arabia

## Abstract

**Objective:**

To estimate the prevalence of self-reported physician-diagnosed obesity and identify its risk factors among adults attending primary healthcare centers in Riyadh, Saudi Arabia.

**Methods:**

A cross-sectional survey was conducted from March to July 2023. Participants aged 18 years or older attending 48 randomly selected primary healthcare centers were recruited using a multistage sampling approach, involving random selection of primary healthcare centers followed by systematic random sampling of attendees. Obesity status was defined based on self-reported prior physician diagnosis of obesity, collected using a validated questionnaire. Multivariable logistic regression was used to identify factors independently associated with obesity.

**Results:**

A total of 14,239 adults participated in the study. The prevalence of self-reported physician-diagnosed obesity was 5.2% (95% CI: 4.9–5.6). Males were 30% less likely to be obese than females (AOR: 0.72; 95% CI: 0.60–0.86), while smokers were more than twice as likely to be obese compared to non-smokers (AOR: 2.37; 95% CI: 1.94–2.89). Fast food consumers higher odds of obesity (AOR: 1.61; 95% CI: 1.24–2.09). Obesity was also positively associated with diabetes (AOR: 1.48; 95% CI: 1.15–1.89), hypertension (AOR: 1.60; 95% CI: 1.23–2.09), hypercholesterolemia (AOR: 4.36; 95% CI: 3.43–5.55), and heart disease (AOR: 4.46; 95% CI: 3.47–5.74).

**Conclusion:**

Self-reported physician-diagnosed obesity was significantly associated with behavioral and cardiometabolic risk factors among adults attending primary healthcare centers. These findings highlight the importance of early identification and integrated management of obesity-related comorbidities in primary care settings.

## Introduction

Obesity has become a global epidemic, largely driven by urbanization, industrialization, and evolving dietary habits (1, 2). As a significant public health challenge of the 21st century, it contributes to a wide range of cardiovascular diseases (3–5). This rise is further fueled by global food system changes, including the adoption of Westernized diets and increasingly sedentary lifestyles due to globalization (6). Beyond these global shifts, socioeconomic and behavioral factors play a key role in obesity’s development, often alongside other non-communicable diseases (7, 8). Obesity substantially increases the risk of morbidity and mortality from conditions like Type 2 diabetes, hypertension, hypercholesterolemia, and metabolic syndrome (9–11). Consistent research links obesity to elevated cardiovascular complications and mortality (5). Disturbingly, evidence also suggests that economically disadvantaged and marginalized communities are increasingly affected by obesity as they embrace Westernized lifestyles (12).

Saudi Arabia has seen a similar increase in obesity, largely due to its shift from traditional to Westernized lifestyles (13). Health surveys in the country show an upward trend in obesity prevalence, particularly among economically disadvantaged children and adults (14). Over the last two decades, this surge in obesity has been driven by decreased physical activity and increased fast food consumption (15, 16). Despite these trends, there remains a relative lack of large-scale, contemporary studies that combine systematic, representative sampling with the assessment of both obesity prevalence and associated comorbidities among adults in Saudi Arabia. Previous studies often relied on convenience samples, were limited to specific regions or subpopulations, or used objective measures without exploring behavioral and sociodemographic determinants (17–19). This methodological gap limits the ability to generalize findings and to identify population-level risk factors that could inform targeted interventions. Accordingly, this study aimed to estimate the prevalence of self-reported physician-diagnosed obesity and examine its associated comorbidities and selected sociodemographic and behavioral factors among adults attending primary healthcare centers in Riyadh, Saudi Arabia.

## Materials and methods

### Study design, timeline, and study duration

We used a cross-sectional survey approach, conducted between March and July 2023. This study was part of the Saudi Arabian Ministry of Health’s (MOH) Health Sector Transformation Program, a key initiative under Saudi Vision 2030. Launched in 2021–2022, this program aims to shift the national healthcare system from a centralized model to a decentralized health cluster model. This transformation focuses on integrating services, improving efficiency, and enhancing patient-centered care. Our research took place in the Riyadh region, specifically within three health clusters managed by Central Health Services. These clusters encompass both primary healthcare centers (PHCs) and hospitals. From the 105 PHCs in the region, 48 PHCs were selected as study sites to facilitate participant recruitment.

### Participant recruitment and sampling strategy

To obtain a representative sample of adults attending PHCs in Riyadh, a multistage sampling approach was applied. In the first stage, 48 PHCs were selected from Health Cluster 2 using stratified random sampling to ensure proportional representation of urban and suburban facilities. Health Cluster 2 was chosen because of its diverse population and strong healthcare infrastructure, serving approximately 3.7 million residents and encompassing 105 PHCs. In the second stage, systematic random sampling was applied at the participant level, whereby every fourth eligible adult attendee in the waiting area of each selected PHC was invited to participate.

Thus, while PHCs served as recruitment sites, participants were sampled systematically from healthcare attendees rather than through population-based cluster sampling, reflecting users of primary healthcare services in Riyadh. Participation was voluntary, and informed consent was obtained prior to data collection. Since study participants were recruited exclusively from PHCs, the study population represents individuals utilizing healthcare services rather than a household-based sample of the general population. As such, findings primarily reflect obesity diagnoses and associated factors among primary healthcare attendees in Riyadh.

### Inclusion and exclusion criteria and sample

Our study included adults aged 18 and over who visited the selected PHCs. This encompassed both Saudi nationals and non-Saudi residents accessing services at these facilities, regardless of their residency status. Conversely, we excluded healthcare professionals and support staff working at the participating PHCs, individuals under 18, patients with cognitive impairments that could affect their understanding of the survey, and anyone who declined to provide informed consent. Ultimately, a total of 14,239 adult PHC attendees were recruited through systematic random sampling and included in the final analysis.

### Sample size calculation

The sample size was calculated to estimate obesity prevalence with adequate precision. Assuming an expected obesity prevalence of 30% based on prior national estimates, a 95% confidence level, and a margin of error of 1%, the minimum required sample size was approximately 8,100 participants. To account for potential non-response and to enable subgroup analyses, a larger sample was targeted. The final sample of 14,239 participants exceeded the minimum requirement and provided sufficient statistical power for prevalence estimation and associated analyses.

### Developing research instrument

Our study utilized a structured questionnaire that was the result of a collaborative effort. The Central Health Services Reform Management Team worked alongside consultants from various regions across Saudi Arabia to create this tool. It was part of a larger national health system reform, aiming to standardize how health perceptions, behaviors, and priorities were assessed across all health clusters in the country. The questionnaire was divided into several sections, each focusing on key areas. It explored participants’ self-assessed health status, asking them to rate their health from excellent to poor. Participants’ overall health status was self-reported. They were asked to rate their health on a five-point Likert scale: excellent, very good, good, fair, or poor. This subjective assessment allowed participants to provide a personal evaluation of their general health, capturing both physical and mental well-being as perceived by the individual. We also gathered information on participants’ health priorities and concerns, as well as various health-related behaviors, including tobacco use, fast food consumption, physical activity levels, and alcohol consumption. Physical activity was assessed subjectively by asking participants whether they engaged in any type of physical activity, with responses recorded as a binary variable (Yes/No). No specific duration, frequency, or intensity thresholds were applied. Additionally, the instrument collected essential sociodemographic data like age, educational background, employment status, gender, and marital status, which was simplified to “married” or “single.” Participants also provided details about their medical history and existing comorbidities, such as cardiac conditions, diabetes, obesity, hypertension, and hypercholesterolemia. Finally, we collected information on insurance coverage and asked participants to self-report their history of obesity (Yes/No). This comprehensive design allowed us to capture a wide array of factors relevant to understanding health outcomes and healthcare use within the study population.

### Outcome definition

Participants were asked to report whether they had ever been diagnosed with obesity by a physician (Yes/No). Thus, the primary outcome in this study was self-reported physician-diagnosed obesity, rather than obesity defined using objectively measured body mass index (BMI). Height and weight measurements were not collected as part of this survey; therefore, BMI-based obesity classification could not be determined.

### Validating and ensuring the research instrument’s reliability

To ensure the high quality of our research instrument, we performed a thorough validation and reliability assessment. Content validity was first established by a panel of 15 experts, comprising healthcare practitioners and public health specialists. They reviewed the questionnaire for its relevance, clarity, and overall suitability for our study objectives, leading to necessary modifications or removals of certain questions to improve its effectiveness. Quantitative assessment of content validity was performed using the Content Validity Index (CVI). Each item was rated by 15 experts on a 4-point relevance scale, and the Item-CVI (I-CVI) was calculated as the proportion of experts rating the item as quite or highly relevant (3 or 4). The scale-level CVI (S-CVI/Ave) was then computed as the average of all I-CVIs, yielding an overall CVI of 0.91, indicating excellent content validity.

Next, we assessed face validity through a pilot study involving 200 participants who were distinct from those in subsequent Hail City pilot testing. These individuals provided feedback on how clear, difficult, and comprehensible the survey questions were. To further aid understanding, our trained data collectors verbally administered the questions during interviews. Reliability was then evaluated using a test–retest procedure with 100 participants from the initial pilot study, completed via telephone after a two-week interval, yielding a test–retest reliability coefficient of 0.83. Finally, to guarantee linguistic accuracy, the questionnaire underwent a rigorous process of translation from English to Arabic, followed by a back-translation to English, ensuring the instrument maintained its accuracy and integrity across both languages.

### Pilot study and justification for Hail City selection

We conducted our pilot study in Hail City, not Riyadh, because the Central Health Services Reform Management Team identified it as an ideal location for preliminary testing. This choice was strategic, as Hail’s demographic and health characteristics were considered to closely mirror those of the wider Saudi population, making it a representative site for initial evaluation. The pilot involved 100 patients and 20 participants in focus group discussions. They provided feedback on the questionnaire’s clarity, comprehensibility, and difficulty. Their crucial insights led to necessary revisions and refinements, ensuring the instrument’s effectiveness before its large-scale implementation in Riyadh and other Saudi health clusters.

### Data collection procedure

Our data collection involved an electronic survey administered by trained data collectors at the participating PHCs. The survey itself was programmed and run on iPads or Android tablets. Before inviting anyone to participate, data collectors verified their eligibility, ensuring only individuals aged 18 or older were included. The survey was conducted through an interview-based approach, where data collectors read questions aloud and directly recorded responses onto the tablets. We collected self-reported data from participants, including whether they had a history of being diagnosed with obesity. This data was not directly linked to patient medical records or specific diagnostic criteria for obesity. Instead, our focus was to identify factors that predict self-reported obesity diagnoses based on sociodemographic, behavioral, and general health information. This method helped us ensure accuracy, minimize missing data, and maintain efficiency. Participants provided information on sociodemographic characteristics (e.g., age, gender, household size, marital status, education level, employment status, and health status), behavioral factors (e.g., tobacco use, fast food consumption and physical activity), and comorbidities (e.g., hypertension, diabetes, hypercholesterolemia, obesity and heart disease). Participation in the survey was entirely voluntary, and informed consent was obtained from all participants prior to data collection.

### Data analysis

Our initial statistical analysis involved examining variable distributions using histograms to check for normality. We summarized normally distributed continuous variables, like age, using means and standard deviations. Age was then grouped into relevant categories to understand participant distribution across different life stages. For categorical variables, including education, employment, marital status, self-rated health, and insurance coverage, we calculated frequencies and percentages to describe the study sample’s characteristics.

We performed univariate logistic regression to find potential predictors for our primary binary outcome, obesity (Yes/No). Variables with a *p* ≤ 0.25 in this univariate analysis were considered for inclusion in the subsequent multivariable logistic regression model. This inclusive criterion helped ensure that all potentially relevant factors were retained for further examination, reducing the risk of missing significant predictors. In the multivariable logistic regression analysis, we systematically evaluated variables to identify characteristics that independently predicted the outcome. We estimated and reported adjusted odds ratios (AORs) and their corresponding 95% confidence intervals (CIs) to quantify the strength and direction of these associations. A *p* < 0.05 was set as the threshold for statistical significance. This approach allowed us to identify independent predictors of obesity while controlling for potential confounding variables. Variable selection for the multivariable logistic regression was guided by both clinical relevance and evidence from prior literature on obesity risk factors, rather than purely statistical criteria (20, 21). Sociodemographic, behavioral, and comorbidity variables identified in previous studies as relevant to obesity were considered for inclusion in the model. While the analysis identifies statistical associations between these factors and self-reported physician-diagnosed obesity, it does not establish causal relationships. Residual confounding due to unmeasured or incompletely measured factors, such as detailed socioeconomic conditions or dietary patterns, may still be present and should be considered when interpreting the results. All statistical analyses were conducted using SPSS version 26.0. The selection of variables for both univariate and multivariable analyses was based on their clinical relevance, observed distribution patterns, and initial associations found during exploratory analyses. This systematic approach ensured a comprehensive evaluation of factors influencing obesity diagnoses within our study population.

Statistical analyses were conducted at the individual participant level using standard logistic regression models. Although participants were recruited from multiple PHCs using a multistage sampling approach, clustering at the PHC level and sampling weights were not explicitly incorporated into the regression models. This approach was adopted because the primary objective was to examine associations between individual-level characteristics and self-reported physician-diagnosed obesity among PHC attendees rather than to generate population-representative estimates. Additionally, participants were systematically sampled within each center, and the inclusion of a large number of PHCs enhanced sample diversity. Nonetheless, the absence of clustering adjustment may result in modest underestimation of standard errors and should be considered when interpreting the precision of estimates.

### Study results

#### Sociodemographic characteristics of study subjects completed the survey

Table 1 details the sociodemographic features with an average age of 59.8 years (±16.35 years). Approximately half of the participants (48.8%) were within the age range of 50 to 75 years, with a further 17.2% being over the age of 75. Males constituted 43.3% of the total sample. Regarding educational attainment, over half of the respondents (51.5%) attended college or university. In terms of marital status, 34.7% of the participants were single, while 51.4% reported being employed. Insurance coverage was reported by nearly a quarter of the participants (24.3%), and 27.7% indicated that they were current smokers. A majority of the participants (60.7%) reported engaging in physical activity, while the prevalence of self-reported physician-diagnosed obesity was 5.2% (95% CI: 4.9–5.6). A relatively small proportion of participants reported having a history of hypertension (11.1%) or diabetes (12.4%).

**Table 1:** Sociodemographic characteristics of study participants completed the survey (*n* = 14,239).

Variables	Frequency (n)	Percentage (%)
Age; mean ± SD	59.78	16.35
Age (years)		
< 50 years	4,848	34
50–75 years	6,945	48.8
At least 75 years	2,446	17.2
Gender		
Female	8,062	56.6
Male	6,177	43.4
Marital status		
Not married	4,939	34.7
Married	9,300	65.3
Education		
Primary	572	4
Up to high school	3,937	27.6
College/University	7,336	51.5
Others	2,394	16.8
Employment status		
Employed	7,317	51.4
Unemployed	6,922	48.6
Health status		
Excellent	4,798	33.7
Very good	5,076	35.6
Good	2,815	19.8
Fair	1,256	8.8
Poor	294	2.1
Insurance coverage		
Yes	3,457	24.3
No	10,782	75.7
Multivitamin use		
Yes	10,131	71.1
No	4,108	28.9
Physical activity		
No	5,598	39.3
Yes	8,641	60.7
Smoking		
No	10,297	72.3
Yes	3,942	27.7
Hypertension		
No	12,659	88.9
Yes	1,580	11.1
Obesity		
No	13,502	94.8
Yes	737	5.2
Diabetes		
No	12,474	87.6
Yes	1765	12.4

#### Sociodemographic predictors of obesity among Saudis at primary healthcare settings in Riyadh

Table 2 presents the sociodemographic predictors of obesity within the Saudi population in this study. As depicted, there appeared to be a trend of increasing obesity with age. The adjusted model revealed that, after accounting for other sociodemographic factors, participants aged 50 to 75 years (AOR: 1.20; 95% CI: 0.96, 1.49) and those aged 75 years and older (AOR: 1.26; 95% CI: 0.95, 1.66) were 1.20 and 1.26 times more likely to be obese, respectively, compared to younger participants (<50 years). Although univariate analysis suggested a strong positive association between age and obesity, this association did not reach statistical significance after multivariate adjustment. Similarly, no significant association was found between education level or employment status and obesity after controlling other factors in the model. However, the multivariable analysis demonstrated that males were approximately 30% less likely to be obese than females (AOR: 0.72; 95% CI: 0.60, 0.86). Married participants showed a slightly higher likelihood of obesity compared to non-married participants (AOR: 1.04; 95% CI: 0.84, 1.28), although this finding was not statistically significant. Notably, study participants with insurance coverage were 1.56 times more likely to be obese than those without insurance coverage (AOR: 1.56; 95% CI: 1.31, 1.85), as shown in Table 2.

**Table 2:** Sociodemographic predictors of obesity among Saudis at primary healthcare settings in Riyadh.

Predictors	Findings of univariate analysis				Findings of multivariable analysis			
		95% CI				95% CI		
	OR	LL	UL	*P*-value	AOR	LL	UL	*P*-value
Age								
< 50 years	1.00			0.07	1.00			0.20
50–75 years	1.18	1.00	1.40		1.20	0.96	1.49	
At least 75 years	1.25	1.01	1.55		1.26	0.95	1.66	
Education								
Primary	1.00			0.01	1.00			0.04
Up to high school	0.60	0.42	0.84		0.64	0.45	0.90	
College/University	0.70	0.51	0.97		0.72	0.51	1.01	
Gender								
Female	1.00			<0.001	1.00			<0.001
Male	0.72	0.62	0.84		0.72	0.60	0.86	
Marital status								
Single	1.00			0.01				0.73
Married	1.22	1.04	1.43		1.04	0.84	1.28	
Employment status								
Employed	1.00			0.21	1.00			0.23
Unemployed	1.10	0.95	1.28		1.12	0.93	1.35	
Insurance coverage								
No	1.00			<0.001	1.00			<0.001
Yes	1.52	1.30	1.79		1.56	1.31	1.85	

OR, odds ratio; AOR, adjusted odds ratio; 95% CI: 95% confidence intervals.

#### Behavioral predictors and co-morbidities linked with obesity among Saudi population

Table 3 illustrates the behavioral predictors and comorbidities associated with obesity within the Saudi population in this study. The results of the multivariable analysis revealed that certain behavioral predictors, namely smoking and fast-food consumption, were significantly related to obesity. Furthermore, comorbidities such as Diabetes Mellitus, hypertension, heart disease, and hypercholesterolemia also demonstrated significant associations with obesity. Specifically, the adjusted model indicated that, after controlling other behavioral risk factors and comorbidities, smokers were 2.37 times more likely to be obese than non-smokers (AOR: 2.37; 95% CI: 1.94, 2.89). The odds of obesity were 1.16 times higher among fast-food consumers compared to those who did not consume fast food (AOR: 1.16; 95% CI: 1.07, 1.26—Corrected CI based on previous interaction). Individuals with diabetes were 1.48 times more likely to be obese than their non-diabetic counterparts (AOR: 1.48; 95% CI: 1.15, 1.89). Additionally, significant positive associations were found between obesity and hypertension (AOR: 1.60; 95% CI: 1.23, 2.09), hypercholesterolemia (AOR: 4.36; 95% CI: 3.43, 5.55), and heart disease (AOR: 4.46; 95% CI: 3.47, 5.74) after adjusting for behavioral risk factors and other comorbidities in the final model, as presented in Table 3.

**Table 3:** Behavioral risk factors and co-morbidities associated with obesity.

Predictors	Univariable analysis			Adjusted for age and sex			Multivariable analysis		
		95% CI			95% CI			95% CI	
	OR	LL	UL	AOR	LL	UL	AOR	LL	UL
Smoking									
No	1.00								
Yes	3.20	2.72	3.76	3.31	2.82	3.90	2.37	1.94	2.89
Physical activity									
No	1.00			1.00			1		
Yes	1.56	1.30	1.86	1.58	1.32	1.89	0.83	0.66	1.04
Fast food consumption									
No	1.00			1.00			1.00		
Yes	1.75	1.42	2.17	1.81	1.46	2.24	1.61	1.24	2.09
Diabetes									
No	1.00			1.00			1		
Yes	4.30	3.61	5.13	4.59	3.82	5.53	1.48	1.15	1.89
Hypertension									
No	1.00			1.00			1		
Yes	6.88	5.79	8.17	7.45	6.22	8.93	1.60	1.23	2.09
Hypercholesterolemia									
No	1.00			1.00			1		
Yes	11.93	10.05	14.17	12.36	10.35	14.76	4.36	3.43	5.55
Heart disease									
No	1.00			1.00			1		
Yes	17.70	14.48	21.65	17.22	14.06	21.07	4.46	3.47	5.74

OR, odds ratio; AOR, adjusted odds ratio; 95% CI: 95% confidence intervals.

#### Multivariable logistic regression analysis: model fitness results

Multivariable logistic regression was used to identify factors independently associated with obesity. Model fit was assessed using the Hosmer–Lemeshow goodness-of-fit test and Nagelkerke R^2^. The Hosmer–Lemeshow test indicated good fit (*p* = 0.46), and the Nagelkerke R^2^ was 0.247, suggesting that the model explains approximately 24.7% of the variance in obesity. The final model converged after six iterations, with parameter estimates stabilizing (change < 0.001).

## Discussion

This cross-sectional survey estimated the prevalence of self-reported physician-diagnosed obesity and examined its associated factors among adults attending primary healthcare centers in Riyadh. Our findings showed that about 5% of participants self-reported as obese. This prevalence is notably lower than what other studies in Saudi Arabia have reported. For example, a 2021 study by Althumiri et al. found obesity in approximately one-quarter of their Saudi Arabian sample (22). Similarly, a 2022 systematic review by Salem et al. on obesity in Saudi Arabia reported a prevalence of about one-third (35.6%) among participants (20).

The markedly lower prevalence observed in our study is likely attributable to the use of self-reported obesity status rather than objective measurements of weight and height. Self-reporting is known to systematically underestimate obesity prevalence, particularly among populations with social desirability concerns, as participants may underreport weight or overreport height (20, 22). In addition, our study population consisted solely of primary healthcare center attendees in Riyadh, who may differ from the general population in health-seeking behavior and self-perception of weight. Previous research has shown that self-reported measures can underestimate obesity prevalence by 20–50% compared with direct measurements (20, 22). Therefore, the low prevalence observed does not necessarily reflect a failure of the measurement tool but rather highlights the limitations inherent to self-reported data. These factors underscore the need for future research to include objective measurements of weight and height to more accurately assess obesity prevalence in the Saudi population.

Because participants were recruited from PHCs, the findings primarily reflect individuals who actively utilize healthcare services rather than the general population. Healthcare attendees may differ systematically from the broader population in terms of age distribution, comorbidity burden, healthcare access, and disease awareness. Individuals who frequently interact with healthcare systems are more likely to have chronic conditions diagnosed, including obesity, whereas individuals who do not routinely access healthcare services may remain undiagnosed. Therefore, the prevalence of self-reported physician-diagnosed obesity observed in this study should not be interpreted as representative of the general population. Nevertheless, these findings remain highly relevant for primary healthcare planning and obesity management, as primary care settings serve as the primary point of diagnosis, prevention, and management of obesity and related cardiometabolic conditions.

We also noted that females were more likely to be obese than males, a pattern seen in other studies globally and within Saudi Arabia. This difference between genders could stem from various factors, such as potentially lower physical activity levels among females and weight fluctuations related to pregnancy. Furthermore, individuals who reported smoking, frequent fast-food consumption, or a diagnosis of diabetes mellitus, hypertension, hypercholesterolemia, or heart disease showed a greater tendency toward obesity compared to those without these factors. These connections align with existing international research and prior studies in Saudi Arabia. For instance, Salem et al.’s 2022 systematic review specifically highlighted how Westernized diets, along with the frequent consumption of fast food and sugary beverages, significantly contribute to the rising obesity rates in the Kingdom (20). Similarly, Althumiri et al. reported a significant association between obesity and type 2 diabetes, hypertension, and hypercholesterolemia within the Saudi population, which closely mirrors the findings of our current study (22).

These consistent findings highlight the strong connection between obesity and several chronic health conditions, underscoring that managing obesity is crucial for addressing these issues in the population. Our results align with extensive global research that has already established the link between obesity and conditions like Type 2 Diabetes Mellitus, hypertension, and hypercholesterolemia (23, 24). Moreover, these findings emphasize the frequent co-occurrence of obesity with other non-communicable diseases (NCDs), which likely increases the overall health burden for Saudi individuals. The simultaneous presence of multiple chronic conditions complicates healthcare management, making it harder for individuals to effectively deal with their health. Therefore, people with multiple comorbidities need targeted interventions and focused attention to improve their health outcomes and lessen the overall impact of disease. Addressing these interconnected health challenges is vital for enhancing quality of life and optimizing health management strategies within this population.

Several established strategies exist for managing obesity and its associated comorbidities. A foundational approach involves lifestyle modifications, encompassing dietary adjustments and increased physical activity (25). In addition to lifestyle modification, pharmacotherapy, and bariatric surgery may also be effective (26–28). Although treatment-related variables were not assessed in this study, these approaches provide important clinical context, as PHCs play a central role in the identification, counseling, and referral of individuals with obesity and related cardiometabolic conditions. The observed associations between self-reported physician-diagnosed obesity and chronic conditions such as diabetes, hypertension, and hypercholesterolemia underscore the importance of early identification and appropriate management within primary care settings. However, the present study does not evaluate treatment effectiveness, and these considerations are provided solely as contextual background.

### Strengths and limitations

This study has several strengths that boost its reliability and how well its findings apply more broadly. We carefully designed the recruitment and sampling methods to match Saudi Arabia’s national demographics and align with past studies in the region. The large sample size and systematic sampling of participants across multiple primary healthcare centers strengthen the internal validity of the study and provide robust insights into obesity diagnoses among primary healthcare attendees. The use of standardized recruitment procedures across 48 centers helped reduce selection bias within the healthcare-attending population. Also, we rigorously pre-tested the questionnaire and assessed both its face and content validity, which makes the collected data more reliable and accurate. The large sample size in our study also significantly contributes to the robustness of the findings, giving us a more precise picture of Saudi Arabia’s diverse adult population.

However, several limitations should be considered when interpreting the findings. The most important limitation is the use of self-reported physician diagnosis to define obesity rather than objective anthropometric measurements such as body mass index (BMI). This approach may result in misclassification bias and likely underestimates the true burden of obesity, as individuals with elevated BMI may remain undiagnosed or unaware of their condition. Self-reported measures are also subject to recall bias and reporting bias. Consequently, the prevalence estimates reported in this study reflect diagnosed obesity rather than BMI-defined obesity and are not directly comparable with national and international prevalence estimates based on objective measurements. Future studies should incorporate direct measurements of height and weight to enable accurate BMI-based classification and improve comparability across populations.

It should be noted that several predictor variables, including physical activity, smoking status, and fast-food consumption, were measured using simplified categorical or binary indicators. For example, physical activity was assessed only as Yes/No without capturing duration, frequency, or intensity. Similarly, smoking and fast-food consumption were categorized broadly without detailed quantification. These simplifications may introduce non-differential misclassification, potentially attenuating the observed associations and reducing the precision of effect estimates. Therefore, while these measures provide useful insight into general behavioral patterns, interpretations regarding their relationship with obesity should be made cautiously and within the context of these measurement limitations.

Additionally, while our multivariable models adjusted for a range of sociodemographic, behavioral, and comorbidity factors, the study design was cross-sectional and not intended to establish causality. The temporal relationship between exposure and outcome cannot be determined, and reverse causality cannot be excluded. Some potentially relevant confounders, such as detailed dietary patterns or socioeconomic nuances, may not have been fully captured. Therefore, the reported associations should be interpreted as correlations rather than causal effects. Furthermore, self-reported behavioral variables, such as smoking status and obesity diagnosis, may be influenced by social desirability bias, potentially resulting in underreporting. Another important limitation relates to the sampling frame. Participants were recruited exclusively from primary healthcare centers, representing a healthcare-utilizing population rather than a true population-based sample. Individuals attending primary healthcare centers may differ systematically from the general population in terms of age distribution, comorbidity burden, health awareness, and healthcare-seeking behavior. Individuals with chronic conditions may be more likely to attend healthcare facilities and receive an obesity diagnosis, whereas healthier individuals or those with limited healthcare access may be underrepresented. This selection bias limits the generalizability of the findings to the broader Saudi population. Therefore, the results should be interpreted as reflecting self-reported physician-diagnosed obesity and associated factors among primary healthcare attendees rather than the general population.

In addition, although participants were recruited from multiple primary healthcare centers using a multistage sampling approach, the statistical analyses did not explicitly adjust for clustering at the primary healthcare center level or apply sampling weights. Failure to account for clustering may result in underestimation of standard errors and narrower confidence intervals, potentially affecting the precision of estimates. However, given the large sample size and inclusion of participants from 48 centers, the impact of clustering is likely modest. Future studies using multistage sampling designs should consider applying multilevel modeling or complex survey analysis techniques to appropriately account for clustering and improve variance estimation.

## Conclusion

This study found a relatively low prevalence of self-reported physician-diagnosed obesity among Saudi individuals attending primary healthcare centers in Riyadh. This lower prevalence likely reflects the use of self-reported physician diagnosis rather than objective BMI-based measurements and should not be interpreted as representing the overall obesity burden in the general population. Despite this limitation, our findings demonstrate significant associations between self-reported physician-diagnosed obesity and behavioral factors such as smoking and frequent fast-food consumption, as well as established comorbidities including type 2 diabetes mellitus, hypertension, and hypercholesterolemia. These findings highlight the important role of primary healthcare settings in identifying individuals with obesity and related cardiometabolic conditions. Early identification and appropriate management of obesity and its associated risk factors within primary care may help reduce the burden of chronic diseases. Given the observed associations with multiple comorbidities, strengthening obesity screening, patient education, and preventive strategies in primary healthcare settings is essential. Future research incorporating objective anthropometric measurements and population-based sampling is needed to provide more accurate estimates of obesity prevalence and to better inform public health strategies in Saudi Arabia.

## Data Availability

The original contributions presented in the study are included in the article/supplementary material, further inquiries can be directed to the corresponding author.

## References

[1] Philip W James T. Obesity—a modern pandemic: the burden of disease. Endocrinol Nutr. (2013) 60:3–6. doi: 10.1016/S1575-0922(13)70015-9, 24490215

[2] Nicholls SG. Standards and classification: a perspective on the ‘obesity epidemic’. Soc Sci Med. (2013) 87:9–15. doi: 10.1016/j.socscimed.2013.03.009, 23631773

[3] Jayawardena R Byrne NM Soares MJ Katulanda P Hills AP. Prevalence, trends and associated socio-economic factors of obesity in South Asia. Obes Facts. (2013) 6:405–14. doi: 10.1159/000355598, 24107686 PMC5644757

[4] Lissner L Visscher TL Rissanen A Heitmann BL. Monitoring the obesity epidemic into the 21st century-weighing the evidence. Obes Facts. (2013) 6:561–5. doi: 10.1159/000357539, 24356471 PMC5644895

[5] Aune D Sen A Norat T Janszky I Romundstad P Tonstad S . Body mass index, abdominal fatness, and heart failure incidence and mortality: a systematic review and dose–response meta-analysis of prospective studies. Circulation. (2016) 133:639–49. doi: 10.1161/CIRCULATIONAHA.115.016801, 26746176

[6] Kopp W. How western diet and lifestyle drive the pandemic of obesity and civilization diseases. Diabet Metab Syndr Obes. (2019) 12:2221–36. doi: 10.2147/DMSO.S216791, 31695465 PMC6817492

[7] Popkin BM Adair LS Ng SW. Global nutrition transition and the pandemic of obesity in developing countries. Nutr Rev. (2012) 70:3–21. doi: 10.1111/j.1753-4887.2011.00456.x, 22221213 PMC3257829

[8] Gupta R Deedwania PC Sharma K Gupta A Guptha S Achari V. Association of educational, occupational and socioeconomic status with cardiovascular risk factors in Asian Indians: a cross-sectional study. PLoS One. (2012) 7:e44098. doi: 10.1371/journal.pone.0044098 22952886 PMC3430674

[9] Dominguez L Galioto A Ferlisi A Pineo A Putignano E Belvedere M . Ageing, lifestyle modifications, and cardiovascular disease in developing countries. J Nutr Health Aging. (2006) 10:143. 16554951

[10] Ali MK Bhaskarapillai B Shivashankar R Mohan D Fatmi ZA Pradeepa R . Socioeconomic status and cardiovascular risk in urban South Asia: the CARRS study. Eur J Prev Cardiol. (2016) 23:408–19. doi: 10.1177/2047487315580891, 25917221 PMC5560768

[11] Leggio M Lombardi M Caldarone E Severi P D'Emidio S Armeni M . The relationship between obesity and hypertension: an updated comprehensive overview on vicious twins. Hypertens Res. (2017) 40:947–63. doi: 10.1038/hr.2017.75, 28978986

[12] Mackenbach JP Stirbu I Roskam A-JR Schaap MM Menvielle G Leinsalu M . Socioeconomic inequalities in health in 22 European countries. N Engl J Med. (2008) 358:2468–81. doi: 10.1056/nejmsa0707519, 18525043

[13] Memish ZA El Bcheraoui C Tuffaha M Robinson M Daoud F Jaber S . Peer reviewed: obesity and associated factors—Kingdom of Saudi Arabia, 2013. Prev Chronic Dis. (2014) 11:E174. doi: 10.5888/pcd11.140236, 25299980 PMC4193060

[14] Nasreddine L Tamim H Mailhac A AlBuhairan FS. Prevalence and predictors of metabolically healthy obesity in adolescents: findings from the national “Jeeluna” study in Saudi-Arabia. BMC Pediatr. (2018) 18:1–15. doi: 10.1186/s12887-018-1247-z 30139344 PMC6107964

[15] Alsulami S Baig M Ahmad T Althagafi N Hazzazi E Alsayed R . Obesity prevalence, physical activity, and dietary practices among adults in Saudi Arabia. Front Public Health. (2023) 11:1124051. doi: 10.3389/fpubh.2023.1124051, 37056656 PMC10086171

[16] Wahabi H Fayed AA Shata Z Esmaeil S Alzeidan R Saeed E . The impact of age, gender, temporality, and geographical region on the prevalence of obesity and overweight in saudi arabia: scope of evidence. Healthcare. 11:1143. doi: 10.3390/healthcare11081143 PMC10137821 37107976

[17] Banany M Gebel K Sibbritt D. An examination of the predictors of change in BMI among 38 026 school students in Makkah, Saudi Arabia. Int Health. (2024) 16:463–7. doi: 10.1093/inthealth/ihae029 38578607 PMC11218882

[18] Alsulami S Althagafi N Hazazi E Alsayed R Alghamdi M Almohammadi T . Obesity and its associations with gender, smoking, consumption of sugary drinks, and hour of sleep among king Abdulaziz university students in Saudi Arabia. Diabet Metabol Syndr Obes. (2023) 16:925–34. doi: 10.2147/DMSO.S405729, 37033397 PMC10075260

[19] Al-Qahtani AM. Prevalence and predictors of obesity and overweight among adults visiting primary care settings in the southwestern region, Saudi Arabia. Biomed Res Int. (2019) 2019:1–5. doi: 10.1155/2019/8073057 PMC6425323 30949511

[20] Salem V AlHusseini N Abdul Razack HI Naoum A Sims OT Alqahtani SA. Prevalence, risk factors, and interventions for obesity in Saudi Arabia: a systematic review. Obes Rev. (2022) 23:e13448. doi: 10.1111/obr.13448, 35338558 PMC9287009

[21] Al-Raddadi R Bahijri SM Jambi HA Ferns G Tuomilehto J. The prevalence of obesity and overweight, associated demographic and lifestyle factors, and health status in the adult population of Jeddah, Saudi Arabia. Therapeut Adv Chron Dis. (2019) 10:2040622319878997. doi: 10.1177/2040622319878997, 31632623 PMC6769201

[22] Althumiri NA Basyouni MH AlMousa N AlJuwaysim MF Almubark RA BinDhim NF . Obesity in Saudi Arabia in 2020: prevalence, distribution, and its current association with various health conditions. Healthcare. 9:311. doi: 10.3390/healthcare9030311 PMC7999834 33799725

[23] Ortega FB Lavie CJ Blair SN. Obesity and cardiovascular disease. Circ Res. (2016) 118:1752–70. doi: 10.1161/CIRCRESAHA.115.306883, 27230640

[24] Alqarni S. A review of prevalence of obesity in Saudi Arabia. J Obes Eat Disord. (2016) 2:1–6. doi: 10.21767/2471-8203.100025

[25] Klein S Sheard NF Pi-Sunyer X Daly A Wylie-Rosett J Kulkarni K . Weight management through lifestyle modification for the prevention and management of type 2 diabetes: rationale and strategies: a statement of the American Diabetes Association, the north American Association for the Study of obesity, and the American Society for Clinical Nutrition. Diabetes Care. (2004) 27:2067–73. doi: 10.2337/diacare.27.8.2067, 15277443

[26] Aderinto N Olatunji G Kokori E Olaniyi P Isarinade T Yusuf IA. Recent advances in bariatric surgery: a narrative review of weight loss procedures. Ann Med Surg. (2023) 85:6091–104. doi: 10.1097/MS9.0000000000001472, 38098582 PMC10718334

[27] Fisher BL Schauer P. Medical and surgical options in the treatment of severe obesity. Am J Surg. (2002) 184:S9–S16. doi: 10.1016/s0002-9610(02)01173-x, 12527344

[28] Fink J Seifert G Blüher M Fichtner-Feigl S Marjanovic G. Obesity surgery: weight loss, metabolic changes, oncological effects, and follow-up. Dtsch Arztebl Int. (2022) 119:70–80. doi: 10.3238/arztebl.m2021.0359, 34819222 PMC9059860

